# Antibiotic Utilization and Its Implications Among Ruminant Farmers and Stakeholders in Sumbawa Regency, Indonesia

**DOI:** 10.1155/vmi/6519659

**Published:** 2024-12-13

**Authors:** Nurul Jannah, Fahrunnisa, Yudith Vega Paramitadevi, Heryudianto Vibowo, Fariz Am Kurniawan, Nurul Amri Komarudin, Aceng Hidayat

**Affiliations:** ^1^Ecosystem, Environment, and Applied Communication Division, Environmental Management and Engineering Study Program, College of Vocational Studies, IPB University, Bogor, West Java, Indonesia; ^2^Sociology Study Program, Faculty of Social and Political Sciences, Sumbawa University of Technology, Sumbawa, West Nusa Tenggara, Indonesia; ^3^Civil and Environmental Engineering Department, Engineering Faculty, Universitas Indonesia, Depok, West Java, Indonesia; ^4^Production Technology Division, Veterinary Paramedic Study Program, College of Vocational Studies, IPB University, Bogor, West Java, Indonesia; ^5^Production Technology Division, Livestock Management and Technology Study Program, College of Vocational Studies, IPB University, Bogor, West Java, Indonesia; ^6^Environmental Engineering Study Program, Engineering Faculty, University of Singaperbangsa Karawang, Karawang, West Java, Indonesia; ^7^Department of Resource and Environmental Economics, Faculty of Economics and Management, IPB University, Bogor, West Java, Indonesia

**Keywords:** antibiotic misuse, antimicrobial resistance, drivers, practice, qualitative study, ruminants

## Abstract

The rise in antimicrobial resistance is a vital concern, and various factors, such as the overuse of antibiotics in agriculture, have contributed to its development and spread. Livestock farmers, veterinarians, and pharmacies are key prescribers of antibiotics for disease prevention, control, and treatment of ruminant animals. A qualitative study in the Sumbawa District examined their awareness, attitudes, and practices concerning antibiotic use, residues, and resistance, underscoring their vital role in tackling this challenge. The study utilized nine key informant interviews, sixteen in-depth interviews, a single focus group discussion, and on-farm observations using semistructured formats and thematic analysis to identify and explore themes. This revealed a common practice of antibiotic self-medication among ruminant farmers in the Sumbawa Regency, driven by limited knowledge and leading to medicine store purchases based on advice from veterinary paraprofessionals or for unregulated self-treatment. Factors contributing to antibiotic misuse include trust in veterinary paraprofessionals, economic limitations, lack of targeted antiresistance programs, and insufficient regulation of antibiotic sales. Enhancing veterinary paraprofessionals' awareness of their ethical duties, launching educational programs for actors, providing financial support for these initiatives, and strict enforcement of regulations by the local government are strategies that could collectively promote responsible antibiotic use and stewardship.

## 1. Introduction

Antimicrobial resistance, often referred to as AMR, represents a significant challenge for public health worldwide [1], particularly in developing countries such as Indonesia. The livestock subsector, which plays a substantial role in Indonesia's economy, with a gross domestic product (GDP) of 257 trillion in 2019, is similarly affected by this problem [2]. The enormous export volume of the livestock subsector in the livestock products group amounted to a staggering 64.07% in 2017 [3]. Both intensive and extensive smallholder farming are crucial for meeting the market demand for quality, yet affordable, animal protein. However, the irrational use of antimicrobials in livestock is concerning [4]. If antimicrobials are not used appropriately, and access to medicines is not controlled, the risk of bacterial resistance increases [5]. Given its global implications, addressing AMR in the livestock subsector is of utmost importance.

Intensive efforts including regulatory enforcement are underway to combat AMR in Indonesia. The country has an AMR National Action Plan (NAP) under the Ministry of Health derived from the World Health Organization (WHO) policy. However, the conceptual framework is yet to be fully established, and extensive monitoring efforts are still pending [6]. The implementation of the Ministry of Agriculture No. 14 of 2017, which prohibits antibiotic use according to the Classification of Veterinary Drugs, and the establishment of the Ministry of Health Regulation No. 28 of 2021, which provides guidelines for the use of antibiotics, has been somewhat effective.

The surge in antibiotic-resistant bacterial infections in humans has prompted the need to explore factors that contribute to resistance [7]. Antimicrobials are extensively used in livestock to prevent, control, and cure diseases [8]. However, the use of antibiotic drugs in farming can lead to human exposure through the consumption of farm products such as milk and meat, which contain antibiotic residues. This poses a significant health risk owing to the potential transmission of antibiotic-resistant bacteria, including *Enterococcus* spp.*, E. coli*, and *Salmonella* spp., which are present in the digestive tract of farm animals [9]. Farmers, in particular, are at risk of exposure to antibiotic-resistant bacteria because of their daily work, as these bacteria can be transmitted through livestock feces, water, soil, and air [10].

Farmers, veterinarians, and veterinary drug retail outlets play a crucial role in the use of antibiotics to treat and control livestock diseases. Furthermore, more basic knowledge is required to effectively address the issue of antibiotic resistance. Providing them with comprehensive and detailed information about antibiotics, antibiotic residues, and antibiotic resistance will likely reduce the exposure of humans to resistant bacteria [11–13]. Seeking additional knowledge about farmers' understanding, recognition, and conduct with respect to antibiotics use (AU), particularly in regions beyond Java [14], is not just a luxury, but a necessity. Therefore, efforts to control AU, monitor resistance, and develop new strategies are needed in extensive livestock areas, such as in the Sumbawa Regency, West Nusa Tenggara (NTB) Province, Indonesia, to improve knowledge in the community, especially awareness of the use of antibiotics according to one's needs or ratios. According to the 2023 Agricultural Census, the number of agricultural business households in the Sumbawa Regency was 77,496, an increase of 12.28% compared to 2013. Together with other regions in Indonesia, namely, East Java and West Java Provinces, NTB Province is known as one of the largest suppliers of cattle. The Sumbawa Regency makes a significant contribution to the ruminant livestock sector, with 38.38% of its labor force engaged in livestock farming [15]. The increase in livestock farming households aligns with the increasing need for veterinary antibiotics (VA).

Both extensive and intensive farming practices, which differ in their management approaches, are utilized in developing countries. To date, qualitative research on the use of antibiotics has largely concentrated on intensive farms [16–19], with identified roles for actors in these settings. However, its role in extensive farming research remains unclear. Consequently, the overview of prior research in this study is based on intensive farming. Sharma et al. conducted a qualitative study on AU in five intensive dairy farming areas in India, involving participants such as farmers, veterinary paraprofessionals, and veterinarians [20]. In Thailand, Lekagul et al. explored AU in intensive pig farming with actors, including farmers, veterinarians, veterinary drug retail outlets, and government officials [21]. This study, however, investigates the roles of various actors, such as farmers, civil service veterinarians, veterinary paraprofessionals, middlemen, and large-scale business farmers in the Sumbawa Regency, as well as their understanding, beliefs, and practices concerning antibiotics, their residues, and resistance to them.

## 2. Method

**Figure 1:**
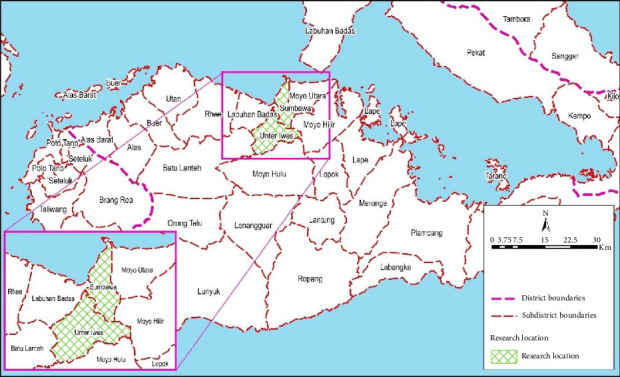
Maps of the Sumbawa subdistrict and Unter Iwes subdistrict showing that the study focuses on the use of antibiotics by ruminant farmers in their livestock area. Created using GIS by Nurul Amri Komarudin.

This research was conducted in Sumbawa Regency (Figure 1), specifically in the Sumbawa and Unter Iwes subdistricts, which host the district's largest ruminant farms. The goal of this qualitative study was to explore the use of antibiotics, their residual impact, and the development of antibiotic resistance in the urban subdistrict of Sumbawa and the rural subdistrict of Unter Iwes. Conducted from January to April 2024, the study used a semistructured interview protocol with open-ended questions, which received ethical approval from the Health Research Ethics Committee of the Faculty of Medicine, Cipto Mangunkusumo Hospital, University of Indonesia (ethics number: KET-1254/UN2/F1/ETIK/PPM.00.02/2023).

### 2.1. Descriptions of Research Participants

This study focused on farmers managing medium-sized cattle and buffalo fattening businesses (ruminant farmers) with herds of more than five but fewer than 10 animals, based on prior research indicating that small farms typically have 2–5 animals [22]. This approach ensures consistent data, informs tailored policies, and supports programs distinct from those for small- or large-scale farms. Respondents were selected from groups involved in the ruminant livestock marketing supply chain in Sumbawa Regency, based on recommendations from local agricultural offices. The respondents, detailed in Table 1, included veterinarians from the “Puskeswan” Animal Health Center, veterinary drug retail with at least a third-level diploma in veterinary paraprofessionals, middlemen or “peleleh” trading cattle and buffalo, and large-scale business farmers. Data were collected through interviews, focus group discussions (FGDs), observations, and photographs.

### 2.2. Data Collection

Key informant interviews (KIIs) were conducted with 10 individuals selected through purposive sampling. Participants included professionals with at least 1 year of full-time experience. The key informants comprised two veterinarians, three veterinary drug retailers, three middlemen, and two large-scale farmers. Veterinary paraprofessionals administer medication under the supervision of veterinarians. Therefore, interviewing veterinarians should cover the topic of medication use.

After conducting KIIs, the research team carried out in-depth interviews to gain a detailed exploration of AU. These interviews involved 16 medium-scale ruminant farmers from the Sumbawa and Unter Iwes subdistricts. The farmers were selected through purposive sampling, with criteria including being at least 18 years old and having a minimum of 2 years of farming experience. All interview transcripts were kept anonymous, with no gender-based differentiation. To ensure that no new information was emerging, and that the data were already saturated [23, 24], the study also conducted a two-hour FGD in Indonesian and *Samawa* languages, with data recorded in written notes and audio recordings.

### 2.3. Analysis and Management Data

The collected data were securely stored in cloud storage with password protection, ensuring only the primary research team had access. Backups were made on a solid-state drive and stored safely. Verbatim transcripts were translated into English and anonymized. Data analyses were conducted using open-source Taguette software (https://app.taguette.org/). The primary investigator created preliminary codes by analyzing all transcripts [25], which were reviewed alongside transcript notes. Two additional researchers helped organize these codes into themes and subthemes. To reduce bias, the research team continuously discussed their findings and interpretations. In qualitative research, a theme is considered novel if it is significant and relevant to the entire dataset [21, 26]. The types of antibiotics used were taken from the transcript of the KIIs. These types were verified through a survey of veterinary drug retail outlet owners. The quantitative data were then analyzed using GraphPad Version 10.0.0.

B.F. Skinner's operant conditioning theory [27] was used to examine attitudes and behaviors related to antibiotic misuse in this study. This theory suggests that behavior is shaped by its consequences. While Skinner's theory has been widely applied in various fields, it has not been specifically developed to address antibiotic misuse. However, its principles offer valuable insights into understanding and potentially modifying behaviors related to this issue. By examining the consequences that shape these behaviors, more effective strategies to combat antibiotic misuse can be developed.

## 3. Results

### 3.1. Key Informant Interview of Stakeholders at the Ruminant Livestock Supply Chain in Sumbawa Regency

Among the 10 KII respondents, only the middlemen (*n* = 3, 33.3%) admitted to not using VA in their daily ruminant care practices. The specific types of VA used are known by five respondents (55.6%), who are veterinarians and veterinary drug retail outlets. Veterinarians prioritize proper vaccination and treatment, and they also educate farmers about the risks associated with antibiotic residues. Veterinary drug retailers provide high-quality medicines and offer consultation services to buyers. Large-scale business farmers integrate traditional and modern methods in livestock maintenance, including the use of traditional herbal remedies. Livestock suppliers rely on their expertise to assess the condition of livestock and use traditional antimicrobials to prevent diseases. The parties involved, namely veterinarians, veterinary drug retail outlets, middlemen, and large-scale business farmers, based on key informant interview analysis, are presented in the following description.

#### 3.1.1. Veterinarian

##### 3.1.1.1. Public Trust in Veterinary Paraprofessionals Compared to Veterinarians

Based on the findings of key informants, including veterinarians, the diseases reported to infect livestock in the two subdistricts were foot and mouth disease, anthrax, haemorrhagic septicemia, pneumonia, bovine ephemeral fever, rabies, diarrhea, lumpy skin disease, dyspepsia, and scabies. In addition, the balische-ziekte skin disease is a noncommunicable disease that often affects livestock owing to the consumption of *L. camara*. Livestock in the extensive system are more susceptible to infectious diseases than those in the intensive system, and animals are frequently found dead along the road because of the late identification of diseases. As previously stated, one of the reasons for the delay in examination by a veterinarian is the practice of farmers providing self-treatment, with the veterinarian being called only after the livestock has an emergency condition or when the veterinary paraprofessional is no longer able to handle the situation.

*Currently, medications are freely accessible to farmers, so they typically handle the situation on their own before seeking assistance from healthcare professionals.*

*Veterinarian 01, Unter Iwes subdistrict.*

The primary reasons farmers seek services from veterinary paraprofessionals to treat their livestock are twofold. First, there was a considerable discrepancy between the number of accessible veterinarians and veterinary paraprofessionals on duty. Second, in the livestock sector, a common belief is that consulting a veterinary paraprofessional is considerably less expensive than engaging a veterinarian. The number of veterinarians available at each animal health center in each subdistrict varied from one to three.

*Farmers are accustomed to calling veterinary paraprofessionals after livestock has been sick for days, making AU difficult for veterinarians to track.*

*Veterinarian 02, Sumbawa subdistrict.*

##### 3.1.1.2. Socialization of Farmers Regarding the Use of Antibiotics, Their Residues, and Resistance Has Not Been Provided by the Authority

The Animal Husbandry and Animal Health Service Office has been raising awareness among ruminant farmers, primarily focusing on hygiene and sanitation programs within the farm environment as well as the proper handling of sick animals resulting from endemic diseases. Annually, the office organizes the register program, a livestock recording activity that provides farmers with the opportunity to engage with health workers. Although the office has previously examined the issue of monitoring drug residues in livestock products, it has yet to specifically address the concepts of antibiotics, residues, and resistance.

*The dissemination of information on drug residues, community farm sanitation hygiene, and meat inspection in relation to the One Health Policy has been limited to a door-to-door approach. However, the socialization of this topic has never been conducted on a large scale.*

*Veterinarian 02, Sumbawa subdistrict.*

#### 3.1.2. Veterinary Drug Retail Outlet

##### 3.1.2.1. Sale of Antibiotics Without Prescription

Owners of medicine stores in the Sumbawa and Unter Iwes subdistricts noted that several brands of broad-spectrum antibiotics were popular among farmers and were frequently sought after. The absence of oversight from relevant authorities has resulted in these antibiotics being regarded as common medications by farmers.

*We recommend the administration of broad-spectrum antibiotics and provide guidance on their proper use in relation to the animal's weight when the farmer arrives. Initially, we inquired about the type of animal and its weight, as the use of antibiotics is associated with potential hazards. A concern exists that animals administered antibiotics and later slaughtered may still have residual antibiotics in their systems, posing a risk to consumers. To mitigate this risk, it is suggested that animals be slaughtered 5 days after administering antibiotics. Therefore, we remind you of this important precaution regarding the use of antibiotics.*

*Veterinary drug retailer 02, Unter Iwes subdistrict.*

#### 3.1.3. Middleman

##### 3.1.3.1. Facilitate the Expedited Sale and Conveyance of Livestock

Middlemen play a crucial role in the livestock supply chain by facilitating sales between farmers and buyers. Despite their function as intermediaries, middlemen can receive substantial payments ranging from USD 32 to USD 200 for each livestock sold. The middleman emphasized that it is mandatory for farmers to conduct thorough health checks on their livestock before they are sold.

*My acquaintances and I typically employ a pickup truck for our transportation needs. With this vehicle, I could carry up to four animals at a time. We frequently transported livestock, but we only selected healthy animals that had not received any medication within the last 5 days. On a monthly basis, we can transport livestock 5-6 times, and we have buyers located in Java and Kalimantan.*

*Middleman 01, Unter Iwes subdistrict.*

The middleman typically possesses a yellow card or road letter to relocate the animal from the veterinary drug retailer's location to the buyer's location. Exhausted cattle and buffaloes may receive traditional remedies, such as *lita bark, malam stick*s, and vitamin B from middlemen. Antibiotics are rarely administered, and consultation with a veterinary paraprofessional or veterinarian is seldom conducted, as the animals remain on the middleman's premises for only 1-2 weeks prior to being sold.

*Cattle transmission has never been an issue. My knowledge in this regard is limited because of the short duration they stay with me. Usually, they are with me for a week or two, or just one night, before being sold. In cases where the price was too low, I slaughtered them and sold the meat in the village.*

*Middleman 03, Sumbawa subdistrict.*

#### 3.1.4. Large-Scale Business Farmers

##### 3.1.4.1. Fodder Supply Limitations and Knowledge Gaps in Antibiotic Use

The land area allocated for cattle and buffalo rearing in Sumbawa Regency typically exceeds 0.5 km^2^ and features permanent pens. These facilities are owned by large-scale business farmers, who also possess grass choppers, stump crushers, corn kernel choppers, and water sources to support livestock feed production. The primary sources of fodder for animals include white leadtree and elephant grass from their own land, whereas corn stalks are occasionally obtained from other local farms. Nonetheless, large-scale business farmers cited a limited supply of fodder as a pressing challenge that could hinder the fattening process of their livestock.

The prevention of livestock diseases is achieved through various methods, depending on the health status of the animals. If animals appear sick, self-medication may be administered. According to the individuals interviewed, antibiotics and herbal remedies were typically obtained from medical stores. Large-scale business farmers admit that most farmers in the Sumbawa Regency rarely seek the services of veterinarians, because they believe that a veterinarian is necessary only when sick animals cannot be treated on their own. During disease outbreaks, large-scale business farmers directly send vaccination requests to veterinarians at the Animal Husbandry and Animal Health Service Office. When questioned about their use of antibiotics, large-scale business farmers claimed to be unaware that antibiotics were the responsibility of the healthcare professionals.

*I do not suggest that the use of antibiotics in animals may have negative effects on human health and the environment. Currently, I do not have knowledge about antibiotic resistance. Does the use of antibiotics and the development of antibiotic resistance not fall under the responsibility of medical professionals or veterinarians?*

*Large-scale business farmers 01, Sumbawa subdistrict.*

#### 3.1.5. Relationships Between Various Parties Involved in Ruminant Farming

**Figure 2:**
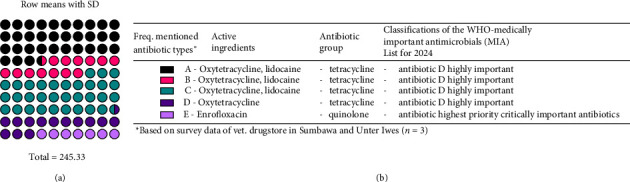
List of antibiotic active ingredients based on an antibiotic survey of veterinary drug retail outlet in the Sumbawa. Regency. (a) The calculation of the difference between the stocked antibiotics and the sold antibiotics from each veterinary drugstore (*n* = 3) revealed that the total difference was 245.33. Antibiotic A had the highest proportion (mean, SD = 82 ± 16.7, 33.42%), while Antibiotic E had the lowest proportion (mean, SD = 16.67 ± 28.87, 6.79%). (b) An explanation of Figure 2(a), including information about the types of antibiotics mentioned in the KII transcript, validated through survey results.

In the subdistricts of Unter Iwes and Sumbawa, sick animals are often treated with Antibiotic A containing the active ingredient oxytetracycline from the tetracycline group, administered through injection or bolus (as shown in Figure 2). Antibiotic A is not only prescribed by official veterinarians but is also used by veterinary paraprofessionals and sold by medicine stores to farmers and large-scale business farmers. The preference for Antibiotic A is due to its long-lasting effect. Antibiotic E, which contains the short-acting active ingredient, enrofloxacin, is reserved for the treatment of pneumonia and is used as a last resort. Veterinary paraprofessionals recommend Antibiotics A to D for farmers and large-scale business farmers, who then obtain them from medical stores. Over the past 5 years, farmers have become familiar with these antibiotics, which they consider common medicines.

### 3.2. In-Depth Interviews of Medium-Scale Ruminant Farmers

#### 3.2.1. Livestock Farming Practices and Challenges in Sumbawa and Unter Iwes Subdistrict

Table 2 presents the characteristics of the 16 farmers ascertained through in-depth interviews. Farmers share a common goal in livestock rearing, treating it as a savings or asset alongside corn farming activities. Cattle and buffaloes are sold once every 6 months. Each medium-sized farmer possesses 0.3–0.5 km^2^ of land that is integrated into their agricultural land. It is uncommon for middle-class farmers to keep their livestock in their own yards, and the distance from their homes to their farms ranges from 2 to 13 km. Farmers were primarily drawn to the profession due to environmental factors, such as growing up in farming families and cultural traditions and personal interests. A significant proportion (62.5%) has been engaged in farming for over 25 years. However, the remaining farmers had commenced farming operations within the last 5 years. The introduction of compensation regulations in certain rural regions has prompted farmers to cease the longstanding practice of “lar,” which involves releasing livestock to graze, due to the financial liability for any damage caused to others' crops by their animals.

Ruminant farmers typically engage in the sale and purchase of livestock through middlemen known as “peleleh,” who visit the farmers monthly to conduct transactions. These transactions occur upon a price agreement, whereas farmers selling directly during the Eid al-Adha season tend to concentrate on fattening the livestock. Generally, there are four main challenges: (1) scarcity of water and nutritious feed during the dry season, (2) extensive land conversion in the Sumbawa District for corn cultivation, (3) prevalence of infectious diseases in livestock, and (4) incidents of livestock theft during land practice.

*The first is the difficulty of finding feed and water for livestock during the dry season. Second, the land for livestock is getting narrower because people have started to plant corn or rent out their land to plant corn, so the land is no longer as free as it was in 2010. Third, my cattle often get a fever for 3 days, and then they become lame, and their hair stands up. Once I could not save my cattle because of an illness. I forgot the name of the disease, and it was treated by a veterinarian but could not be saved.*

*Farmer 16, Unter Iwes subdistrict.*

Farmers primarily adopted the farrow-to-finish approach, with new farmers who had been in service for less than 10 years providing concentrates in addition to forage for their animals one to two times per day (Table 2). Conversely, farmers with more than 25 years of experience opted to feed their animals exclusively on forage daily.

#### 3.2.2. The Widespread Use of Drugs and Antibiotics Without a Veterinarian's Prescription

As shown in Table 2, medicines in the form of concoctions without patent brands, herbs, or antibiotics are commonly administered by farmers when livestock suffers from illnesses. Farmers commonly use medicines and antibiotics. As a preventative measure to maintain livestock health and prevent diseases, farmers with more than 25 years of experience (62.5%, 10/16) commonly administer traditional remedies to their livestock. Farmers with less than 10 years of experience tend to buy vitamins and minerals from medicine stores as a preventive measure. Farmers often purchase herbal remedies, pharmaceuticals, and antibiotics from veterinary drug retail outlets to independently treat livestock illnesses. They also reported that those medicines and antibiotics were readily available for purchase at veterinary drug retailers without restrictions or prohibitions.

*…indeed, we sometimes receive herbs from animals or purchase them. Occasionally, we create our own concoctions, such as turmeric water, to boost their appetite, incorporating peeled bark. I utilize lita bark, moringa bark, salt, and turmeric. In addition, I acquired a product from a store that was reputed to enhance the cows' appetite.*

*Farmer 05, Sumbawa subdistrict*

It is common for farmers to purchase herbal concoctions and antibiotics from medicine stores based on the advice of fellow farmers, relatives with veterinary degrees, and veterinary aides. Participants indicated that the reasons for buying drugs and antibiotics included fear of disease impact, easy access, choice of products based on popular brands, and a lack of sales restrictions on antibiotics. In cases where livestock has been ill for several days or in emergencies, farmers tend to seek the services of a veterinary paraprofessional (9/16, 56.25%) or veterinarian (7/16, 43.75%).

*If my livestock(s) get sick, I usually buy medicine from the store. I treat myself. If it is an emergency, then I get the veterinary paraprofessionals involved.*

*Farmer 01, Sumbawa subdistrict.*

*The last time I called the veterinarian from Kebayan (-name of the place) was last year (2023). At that time, my cow had a skin disease. In addition, there were large swollen wounds. He diagnosed it as foot-and-mouth disease.*

*Farmer 02, Sumbawa subdistrict.*

Farmers rarely receive regular consultations from health workers other than through the initial registration of livestock; therefore, they do not have a plan to care for their livestock population. Farmers in the Sumbawa and Unter Iwes subdistricts have the same characteristics when prescribing antibiotics for their own livestock, namely, successful treatment of diseases that are perceived to have similar symptoms in the past, passing down from parents, advice from fellow farmers, and relatives or veterinary paraprofessionals.

*When local farmers, who are my friends, gather, we often talk extensively about cattle and share our experiences. It is rare to have veterinarians or veterinary paraprofessionals around. However, when they visit to check on our cattle, we seize the opportunity to ask questions and engage in conversations with them.*

*Farmer 14, Unter Iwes subdistrict.*

#### 3.2.3. Lack of Comprehension and Acknowledgment Among Farmers About Antibiotics and Their Resistance

Farmers who purchase antibiotics from medical stores often lack an understanding of these medications and rely on veterinary drug retail outlet instructions or label guidelines. Nonetheless, farmers received broad-spectrum antibiotics for no clinical reasons. Farmers with over 25 years in the field have been known to administer antibiotics directly to their animals.

*...I understand its application. It varies according to the age of cows. Typically, those prone to illness range from 6 months to a year old. We administered 3* *cc of the antibiotic to a 6 month-old infant. For a 1 year-old, we administered 6* *cc...*

*Farmer 03, Sumbawa subdistrict.*

*I learned how to inject cattle from my uncle and observed the practices of the veterinary paraprofessionals from the past. For instance, we administered 10 cc of a specific antibiotic to cattle.*

*Farmer 04, Sumbawa subdistrict.*

Many farmers do not understand the concept of AMR, believing that their longstanding livestock practices do not impact the health of animals, humans, or the environment. They believed that the administration of antibiotics accelerates recovery from diseases in livestock, which they find profitable. This is due to the requirement for farmers to certify that their animals are healthy and drug-free for at least 5 days before selling them to buyers. Nonetheless, they are often unaware of the potential for antibiotic residues to remain in meat following treatment.

*I am not familiar with antibiotics specifically, but I typically acquire information about the medications I purchase from acquaintances or hospital staff. If I am unsure how to use them, I seek guidance from the same sources. ...Consultation with a veterinary paraprofessional is typically free of charge; however, for injections, the cost ranges from 50,000 to 100,000 IDR. After receiving the injection, the livestock generally showed improvement the following day.*

*Farmer 07, Sumbawa subdistrict.*

### 3.3. FGD of Medium-Scale Ruminant Farmers

#### 3.3.1. Insufficient Institutional Support From Relevant Authorities

Farmers often face significant challenges due to insufficient institutional support from relevant authorities. Many farmers report a lack of regular visits and consultations from veterinarians, which are crucial for maintaining livestock health. The absence of consistent guidance and education on the use of antibiotics and other medications leaves farmers reliant on informal sources of information, such as fellow farmers or veterinary drug retailer employees. This gap in support is further exacerbated by the lack of awareness and training on critical issues such as antibiotic resistance and proper livestock management practices.

Farmers were not exposed to any form of education or training concerning the use of antibiotics, their residues, or resistance. Their interactions with relevant authorities were limited to once a year during the annual livestock registration program. The topic of antibiotics was not addressed during the induction process, when they joined the livestock association. Furthermore, there is a notable deficiency in the dissemination of regulations and policies related to livestock health, which hinders farmers' ability to comply with best practices and legal requirements.

*There was socialization by the Sumbawa District Livestock Service Office in 2023. That was the moment (program) register. Foot and mouth disease was rampant at the time. For example, what they said; we are advised to separate the place between sick animals and healthy animals so that there is no transmission ... However, for antibiotics, we have never been informed through (the) Register program.*

*Farmer A, during the FGD.*

## 4. Discussion

**Figure 3:**
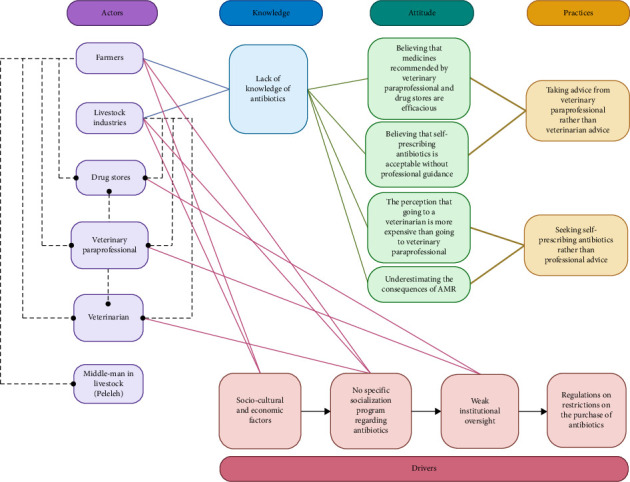
Triggering factors related to actors and their cognitive processes, behaviors, and customs in the research locations. Created by using BioRender by Yudith Vega Paramitadevi.

The intricate nature of the actors' roles in this qualitative study was examined by delving into their knowledge, attitudes, and behaviors, as well as the factors that prompt the use of antibiotics, as stated by Kenyon and Manoharan-Basil [28] (Figure 3). Individuals involved in this scenario had diverse opinions regarding antibiotics. A noteworthy observation is that in the past 5 years, farmers have held the belief that the antibiotics they typically utilize are not considered prescribed medicine and do not require monitoring. The large-scale business farmers share this perspective. In Sumbawa District, antibiotics with similar compositions and active ingredients are widely used. Based on the findings of this study, a research conducted in Kiambu, Kenya, also discovered that Product A, which has gained significant popularity among farmers in the past year, is widely used [26]. The use of antibiotics in the Sumbawa Regency was influenced by a range of sociocultural and economic factors, as well as the lack of a program specifically addressing AMR and insufficient supervision (Figure 3).

### 4.1. The Cultural and Economic Factors Influence the Willingness of Farmers to Seek Antibiotics Prescription From Nonprofessional Sources

Farmers in Sumbawa Regency have traditionally sought treatment for their livestock from veterinary paraprofessionals rather than veterinarians. This practice is fostered and reinforced by positive feedback from family members and fellow farmers. This concept aligns with the findings of Ilukor and Birner's study, which indicated that farmers often continue to rely on veterinary paraprofessionals because of the trust established over time [29]. Even if the advice provided by a veterinary paraprofessional leads to negative consequences, such as the death of animals, they continue to use their services. This contrasts significantly with practices in developed countries, where farmers typically seek guidance from veterinarians regarding the management of diseases in their livestock [30]. In developed nations, the decision-making process regarding livestock is primarily influenced by veterinarians rather than farmers, as is customarily the case in developing countries [31].

Economic factors appear to be primarily influenced by the culture of trust within the farming community and daily interactions with veterinary paraprofessionals. Farmers typically consult veterinary paraprofessionals and medicine stores for various reasons, including the fact that the consultation fee per visit for a veterinary paraprofessional is only a quarter to half of the fee for a veterinarian. In addition, semiextensive farmers in Sumbawa District face pressure from tight profit margins as they are required to share profits with middlemen. However, a study conducted in Uganda suggested that economics is not the primary factor influencing the selection of veterinary paraprofessional services. Small- and medium-sized farmers often lack the ability to distinguish the quality of veterinary paraprofessional work from that of veterinarians [29]. Veterinary paraprofessionals in Sumbawa District have the authority to prescribe antibiotics and administer injections to address the health issues of livestock. Capacity building by veterinary paraprofessionals and their ethical awareness as veterinary assistants could be intervention options, as shown in studies conducted on veterinary paraprofessionals in India [32] and Namibia [33]. Veterinarians in Sumbawa Regency must develop strong connections with veterinary paraprofessionals and farmers to facilitate communication, foster trust, promote collaboration, and enable ongoing learning. Based on McKernan et al.'s review, this collaboration can help veterinarians monitor AU, thereby simplifying antibiotic tracing [31].

### 4.2. The Possibility That Specific Antibiotic Programs May Not Effectively Address the Lack of Antibiotics Knowledge, Leading to Continued Improper AU Despite Their Implementation

Both medium-scale and industrial farmers often lack comprehensive knowledge of the appropriate use of antibiotics, antibiotic resistance, and the potential for antibiotic residues in their products. The infrequent contact between farmers and veterinarians, which usually occurs annually, along with a lack of educational initiatives on the appropriate use of antibiotics, has led to the misconception that antibiotics are over-the-counter medicines in the agricultural sector. This situation poses significant risks such as the emergence of antibiotic resistance.

By BF Skinner's operant theory, it is believed that the antibiotics recommended by veterinary paraprofessionals and medicine stores lead to the immediate relief of symptoms (positive reinforcement), thereby prompting the repetition of such behavior. Conversely, if misuse leads to negative consequences, such as side effects or antibiotic resistance, this behavior tends to diminish. Thus far, these negative consequences have not manifested immediately, have not adversely affected the environment, or are not directly visible to individuals.

Moreover, the knowledge of farmers regarding antibiotics has not yet fully evolved, which leads to predictable attitudes and behaviors when dealing with sick livestock. This situation is similar to that observed in the studies conducted in Kenya and India. In Kenya, 80% of farming households utilize antibiotics for the treatment of their livestock and 58% purchase their own antibiotics from medicine stores [34]. According to research conducted in India, only 49.5% of farmers have knowledge of AMR, and they persist in acquiring antibiotics without a physician's prescription for livestock treatment [16]. This study did not assess the degree of knowledge relative to the educational level of the subjects because antibiotic misuse occurs regardless of their educational background [35]. When intervening, it is imperative to inform all actors about the potential misuse of antibiotics, the need to regulate antibiotic sales, and the development of new drugs, regardless of their educational background. Successful public education initiatives from other countries, such as Japan's characterization approach, Finland's game-based approach, and Switzerland's, can serve as examples of effective implementation [36]. When there is recognition that the misuse of and resistance to antibiotics can cause harm to humans, animals, and the environment, individuals who engage in self-medication may experience self-regulation [37] or societal pressure that results in self-imposed “punishment” [38].

### 4.3. Inadequate Regulation or Supervision Contributes to the Prevalence of Self-Medication With Antibiotics Without Professional Guidance Among Individuals Involved in VA Use

The current situation in the Sumbawa Regency, where antibiotics are readily available in medicine stores and easily accessible, can be attributed to inadequate regulations and sanctions. Drawing from BF Skinner's theory, it is possible to control self-medication behavior by promoting a new understanding that replaces the existing one. As discussed previously, continuous public education can replace outdated knowledge. Public awareness can be heightened through public education, leading to changes in attitudes and behaviors, albeit at a slow pace [39–41]. In accordance with the perspective of Biesheuvel et al., sustainable behavior change requires the incorporation of interdisciplinary elements. This suggests that individuals with expertise in various fields who have successfully applied evidence-based interventions must be involved in the program [30]. Financial support from government and private entities is necessary to ensure the long-term success of public education.

Moreover, the habit of farmers acquiring antibiotics without a prescription remains unchecked because of the scarcity of incentives or restrictive measures (punitive measures). In Indonesia, there is a regulation pertaining to restrictions on antibiotic acquisition, specifically the Ministry of Health Regulation No. 28 of 2021, outlining the guidelines for AU. In accordance with the Sumbawa Regent Regulation 32/2015 about Animal Medicines and Animal Medicines' Business Licenses, measures have been established at the district level. Unfortunately, there was no oversight from Animal Husbandry and Animal Health Services regarding farmers' self-medication practices. In other developing countries, successful outcomes have been achieved through strict enforcement of regulations coupled with public education and other strategies to control self-medication [42, 43]. Proposals proposed by Ferdiana et al. and Howard et al. offer incentives for pharmacies to adhere to regulations and refrain from selling antibiotics without a prescription [44, 45]. Alternatively, synergistic efforts were made by Wulandari et al. to provide special training to medicine store employees so that they could understand the impact of antibiotic resistance so as not to provide services without a prescription [46].

## 5. Conclusion

This study investigated antibiotic usage patterns in ruminant farming within the Sumbawa Regency, Indonesia, to pinpoint cultural, economic, and informational influences on antibiotic administration. Farmers often resort to self-medication or consult veterinary paraprofessionals because of their trust, accessibility, and costs, leading to potential misuse. This study underscores the role of public education and regulatory enforcement in critical intervention. A collaborative, multidisciplinary approach with all actors is essential to address AU complexities. Moreover, regulatory oversight and incentives are vital for curbing self-medication and fostering AU in veterinary practices. A limitation of this study lies in its geographical scope, which may not fully represent the diversity of the Sumbawa Regency. Thus, future studies should extend to more rural, livestock-dense areas for a broader understanding.

## Figures and Tables

**Table 1:** List of respondents in the study of antibiotics, their residues, and resistance in Sumbawa District.

Data collection method	Respondents	Total	Age (years old; mean, range)	Gender man	Gender woman	Latest education
Key informant interviews	Civil servants' veterinarian	2	39; 38–40	—	2	Veterinarian
	Veterinary drug retail outlet	3	51; 42–59	2	1	Associate-master
	Middleman	3	37; 27–48	3	—	High school
	Large-scale business farmers	2	41; 36–45	2	—	Bachelor
In-depth interviews	Medium-scale ruminant farmers	16	49; 23–62	14	2	High school-master
One-time focus group discussion	Medium-scale ruminant farmers	8	49; 30–62	8	—	High school-bachelor

**Table 2:** Characteristics of farmers studied for antibiotics and concoctions/herbs in Sumbawa Regency.

Farmer ID	Number of ruminants		Location and land ownership	Length of time working as a farmer (years)	Use of antibiotics (yes/no)	Frequency of concentrate feed per day	Concoctions/herbs (other alternatives)
	Total cattle	Total buffalo					
Farmer 01	5	2	Sumbawa, private ownership	8	Y	One time	Mixed honey with salt
Farmer 02	5	3	Sumbawa, private ownership	7	Y	One time	Mixed *lita (Alstonia scholaris)* bark and moringa branches
Farmer 03	5	3	Sumbawa, private ownership	41	Y	—	Mixed turmeric with salt
Farmer 04	5	5	Sumbawa, private ownership	42	Y	—	Mixed turmeric, rounds, and bark
Farmer 05	5	1	Sumbawa, private ownership	5	Y	Twice	—
Farmer 06	5	2	Sumbawa, private ownership	5	Y	Twice	—
Farmer 07	5	5	Sumbawa, private ownership	6	Y	Twice	—
Farmer 08	5	2	Sumbawa, private ownership	25	Y	—	Herbal concoctions
Farmer 09	5	1	Unter Iwes, private ownership	26	Y	—	Herbal concoctions mixed with salt
Farmer 10	5	3	Unter Iwes, private ownership	25	Y	One time	Herbal concoctions mixed with salt
Farmer 11	5	1	Unter Iwes, private ownership	28	Y	—	Herbal concoctions
Farmer 12	5	2	Unter Iwes, private ownership	26	Y	One time	Herbal concoctions
Farmer 13	5	3	Unter Iwes, private ownership	27	Y	One time	Herbal concoctions
Farmer 14	5	2	Unter Iwes, private ownership	25	Y	One time	Mixed *lita (Alstonia scholaris)* bark, moringa bark, salt, and turmeric; herbal concoctions
Farmer 15	5	1	Unter Iwes, private ownership	6	Y	twice	Fermented sugarcane bagasse water
Farmer 16	5	2	Unter Iwes, private ownership	35	Y	—	Mixed *lita (Alstonia scholaris)* bark, papaya root, and crushed turmeric

## Data Availability

The data that support the findings of this study are available from the corresponding author upon reasonable request.
